# Do observers like curvature or do they dislike angularity?

**DOI:** 10.1111/bjop.12132

**Published:** 2015-04-13

**Authors:** Marco Bertamini, Letizia Palumbo, Tamara Nicoleta Gheorghes, Mai Galatsidas

**Affiliations:** ^1^ Department of Psychological SciencesUniversity of LiverpoolUK; ^2^ Department of Psychology, Sociology & PoliticsSheffield Hallam UniversityUK

**Keywords:** aesthetics, visual preference, curvature, complexity

## Abstract

Humans have a preference for curved over angular shapes, an effect noted by artists as well as scientists. It may be that people like smooth curves or that people dislike angles, or both. We investigated this phenomenon in four experiments. Using abstract shapes differing in type of contour (angular vs. curved) and complexity, Experiment 1 confirmed a preference for curvature not linked to perceived complexity. Experiment 2 tested whether the effect was modulated by distance. If angular shapes are associated with a threat, the effect may be stronger when they are presented within peripersonal space. This hypothesis was not supported. Experiment 3 tested whether preference for curves occurs when curved lines are compared to straight lines without angles. Sets of coloured lines (angular vs. curved vs. straight) were seen through a circular or square aperture. Curved lines were liked more than either angular or straight lines. Therefore, angles are not necessary to generate a preference for curved shapes. Finally, Experiment 4 used an implicit measure of preference, the manikin task, to measure approach/avoidance behaviour. Results did not confirm a pattern of avoidance for angularity but only a pattern of approach for curvature. Our experiments suggest that the threat association hypothesis cannot fully explain the curvature effect and that curved shapes are, *per se*, visually pleasant.

## Background

People have a preference for curved versions of objects or abstract shapes. Curved shapes tend to be described as more beautiful and more pleasant. This phenomenon is known to artists and to scientists, but its origin is unclear (Silvia & Barona, 2009). The term curvature here refers to smooth changes of curvature along contours, as opposed to abrupt changes (curvature discontinuities), such as the angles of a polygon. We report a series of studies with abstract shapes to examine both explicit and implicit responses, and to investigate whether the effect is driven by a negative response to angularity or a positive response to curvature. In the next section, we review the evidence in favour and against these two possibilities.

### People like curves: The line of beauty

**Figure 1:**
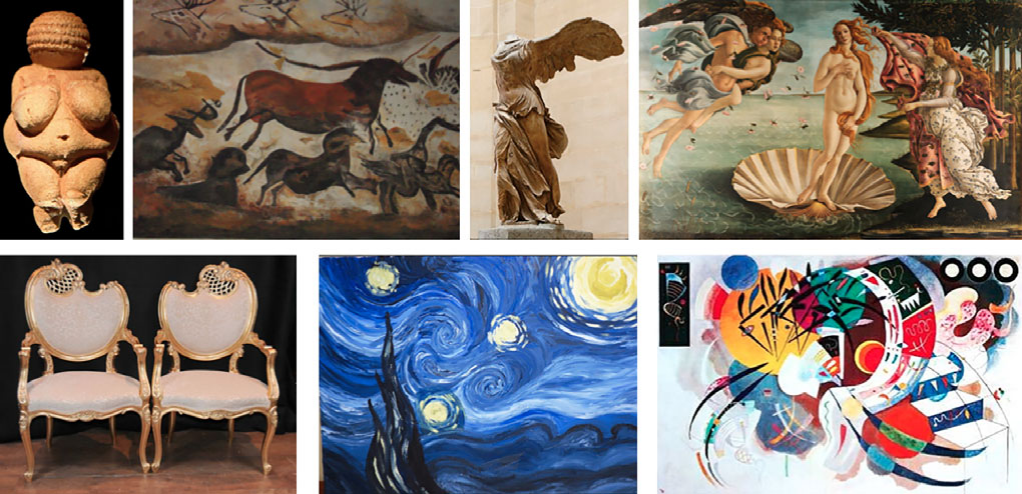
Examples of works of art in which curvature appears to play an important role. From top left: Venus of Willendorf (approximately 26,000 B.C.E.); horses in the Lascaux cave (approximately 17,000 B.C.E.); Nike Statue of Samothrace (190 B.C.); Birth of Venus by Botticelli (1486); the French Rococo Louis XV style armchairs; Starry Night by Vincent Van Gogh (1889); and Dominant Curve by Wassily Kandinsky (1936).

To discuss the preference for curved objects, let us briefly consider visual art. The use of curved lines and shapes spreads across time and cultures. Figure 1 shows a selection of examples from different periods. Curves, waves, and spirals are present in prehistoric statuettes and cave paintings (Clottes, 2003). The second image in Figure 1 is part of the famous Hall of bulls in the Lascaux cave (approximately 17,000 B.C.E.). The bodies of the horses are characterized by curved lines. Because the overall length of the panel is over 4 m, the artist must have produced the curves with multiple strokes. In historical times, curves can be seen in ancient Greek art, as expressed in the wings of the Nike Statue of Samothrace (190 BC) or later during the Renaissance in the shell of the Birth of Venus by Botticelli (1486). More recently, Wassily Kandinsky celebrated curves in his Dominant Curve painting (1936), and Vincent Van Gogh made the use of curves a unique and distinctive brushing technique.

**Figure 2:**
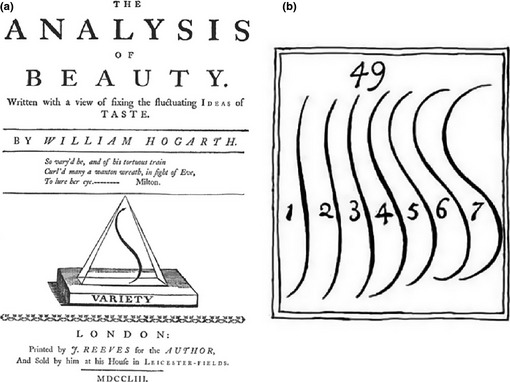
(a) The front cover of ‘The analysis of Beauty’ by William Hogarth (1753). The cover shows the curve defined by the author as the line of beauty. (b) About the lines on the right, Hogarth says ‘Though all sorts of waving‐lines are ornamental, when properly applied; yet, strictly speaking, there is but one precise line, properly to be called the line of beauty, which in the scale of them is number 4: The lines 5, 6, 7, by their bulging too much in their curvature becoming gross and clumsy; and, on the contrary, 3, 2, 1, as they straighten, becoming mean and poor’ (Chapter 10 [49]).

One artist who set out to write a theory about why curves were central to aesthetics is William Hogarth (1697–1764). He was influenced by the Rococo style of the 18th century, which favoured elaborate ornamentation. The name (Rococo) derives from the French words for stone (*rocaille*) and shell (*coquilles*), which is a distinctly curved object. Hogarth detailed his theory in the book ‘The Analysis of Beauty’ published in 1753 (Figure 2). He was interested in beauty as it exists in nature, and not just in a type of cultural phenomenon. The most important objects in his analysis were solid shapes, like the human body, as described by lines. In Chapter VII, he distinguishes lines as *straight*,* curving*,* waving*, and finally as the *serpentine* line which combines waving and curving. In his own words:

For as among the vast variety of waving lines that may be conceived, there is but one that truly deserves the name of *the line of beauty*, so there is only one precise serpentine line that I call *the line of grace*

(Chapter 10, [52]).

Within experimental psychology, one can link the preference for curvature to the principle of good continuation, and therefore of good Gestalt (Kanizsa, 1979). Perhaps a curve is simply a set of strongly grouped points. We will return to the issue of perceptual organization and fluency later. The Gestalt school was also interested in secondary and tertiary qualities of objects (Metzger, 1941; van Tonder & Spehar, 2013). Tertiary qualities concern value and emotion, such as when a face appears *friendly* or *threatening*. They argued that these qualities belong to the stimulus as much as to the observer. Koffka (1935) used the term *physiognomic* properties. The most relevant example from the Gestalt tradition is the ‘takete/maluma’ phenomenon. Köhler (1947) noticed that observers associate angular shapes with the word ‘takete’ and curved shapes with the word ‘maluma’. This association is automatic (Makovac & Gerbino, 2010), and it is part of a semantic network, including a link with pleasant/unpleasant concepts (Milan *et al*., 2013).

Early studies focused on how straight and curved lines were described. Both Lundhom (1921) and Poffenberger and Barrows (1924) found that angular stimuli were associated with terms such as *agitating*,* hard*, and *furious*, and curved stimuli with *gentle*,* sad*,* quiet*, and *lazy* (see also Hevner, 1935). Note, however, an ambiguity in this pattern: Both positive and negative terms are associated with curvature. In another early study, a preference for curvature was reported in typography: Round letters were seen as more pleasant than angular letters (Kastl & Child, 1968). In product design and industry, there are examples of trends towards more smoothed shapes. Leder and Carbon (2005) pointed out that car design has moved away from the straight lines of the late ‘1980s and 1990s’. They studied the role of curvature using car interior design and confirmed that the more curved interiors were more attractive.

The study by Silvia and Barona (2009) investigated similar questions to those dealt with in this study. They asked whether preference for curvature was related to angularity and perceived threat, and how curvature related to complexity. Similar to us, they focused on abstract geometrical shapes. They confirmed a preference for curvature using circles and hexagons, but only for non‐experts. However, when they used irregular polygons a preference for curvature occurred only with expert participants, and this effect remained after controlling for subjective complexity.

Another important study was conducted by Bar and Neta (2006). They used pairs of images depicting everyday objects or meaningless novel patterns. The items in each pair differed exclusively in terms of curvature. Curved objects were preferred not only when compared to sharp‐angled objects, but also when compared to control objects containing both curved and sharp‐angled elements. The universality of a preference for curvature has recently been supported by a cross‐cultural comparison. Gómez‐Puerto, Munar, Acedo, and Gomila (2013) replicated the effect in Spain and with a Ghanaian population.

A few studies provide developmental evidence. Quinn, Brown, and Streppa (1997) reported that 3‐ to 4‐month‐old infants show a spontaneous preference for curvature; thus, visual preference for curved contours seems a fundamental aspect of how humans see the world. Jadva, Hines, and Golombok (2010) confirmed a preference for curved toys over angular ones in 3‐year‐old toddlers.

### Problems with the evidence that people like curves

Although many works of art incorporate smooth curves, and William Hogarth provided a theoretical framework for this, there are also artists who focused on geometrical shapes. For example, *Islamic art* from the 7th century onwards provides examples of geometrical patterns that combine both angular and curved elements, and in the 20th century, *Geometric abstraction* made large use of lines, squares, and corners. The most famous painter in this school is probably Mondrian (1872–1944), and in his mature production, we find a complete absence of curvature.

From experiments using rating or forced‐choice tasks, it is impossible to know whether observers display a preference for curvature or a dislike for angularity. The use of real objects is problematic because of a risk that changes in curvature are related to prototypicality. To address this, Silvia and Barona (2009) confirmed the curvature effect while controlling for symmetry and typicality. However, in their data there was a complex modulation by expertise (i.e., training in visual art), including the absence of an effect when using circles and hexagons with experts. This suggests that preference for abstract shape changes with experience. It is possible that circles and hexagons are shapes that have their own valence due to familiarity. Unfamiliar shapes are preferable, and the reason why Silvia and Barona (2009) moved to random polygons in their second study.

### People dislike angles: Corners are dangerous

Bar and Neta (2006) suggested that the preference for curvature originates from a negative response to angular objects. They argued that the curvature of the contour is a key visual primitive that enables the formation of rapid impressions and that angularity triggers a sense of threat. There is neuropsychological evidence in support of this proposal. Bar and Neta (2007) combined the experimental design used in Bar and Neta (2006) with functional imaging (fMRI) and measured activity in the amygdala, a brain area also involved in processing fear (Ledoux, 2003; Morris, Ohman, & Dolan, 1999). They confirmed the preference for curved objects and found greater bilateral activation in the amygdala for sharp‐angled objects as compared to curved objects. The increased activation in the amygdala was triggered by the presence of sharp‐angled stimuli, and not by the liking response. The authors concluded that angles are features automatically associated with threat. However, the amygdala activation is not specific to fear or negative emotions. There is evidence that the amygdala is also involved in processing positive valanced stimuli (Garavan, Pendergrass, Ross, Stein, & Risinger, 2001) or attractiveness for faces (Winston, O'Doherty, Kilner, Perrett, & Dolan, 2007).

There is also evidence that angles attract attention. Larson, Aronoff, and Stearns (2007) found that downward‐pointing V shapes were detected faster and with greater accuracy than upward‐pointing ones. They suggested that downward‐pointing V are perceived as threatening and thus capture attention. Moreover, an fMRI study by Larson, Aronoff, Sarinopoulos, and Zhu (2009) found that the downward‐pointing V shapes activated the neural networks for threat detection, including the amygdala.

Leder, Tinio, and Bar (2011) have explored the curvature preference by introducing divergent emotional valence to visual stimuli. They used pairs of images with positive or negative emotional valence, such as teddy bears and snakes. The items forming a pair differed solely in the angularity of their contour. People preferred the curved versions of objects to the angular versions, but only if the objects had a neutral or a positive emotional valence. There were no differences in liking for objects with negative emotional valence. Therefore, the emotional valence has precedence over contour in terms of preference.

### Problems with the evidence that people dislike angles

If angles signal threats, one would expect a negative response to shapes that mix angular parts with rounded parts when the angles are on the outside. Some of the control stimuli in Bar and Neta (2007) had this mix of features, but they did not behave as strictly angular stimuli. More importantly, one would predict that as angles become sharper, and thus more dangerous, preference should monotonically decrease. There is no evidence of such a modulation, and instead, there is some evidence of the opposite phenomenon from two studies.

Although Phillips, Norman, and Beers (2010) did not set out to study curvature, their results are relevant. They asked preferences for abstract 3D shapes that varied in complexity. This was manipulated by changing the standard deviation of vertex distances, thus going from a curved blob to an articulated object, which appeared spiky. Observers preferred either very simple or very complex objects. It appears that preference for curved lines does not translate into a general preference for the simplest or the smoothest object, nor for the intermediate level of complexity. The spikiest object was in fact the one selected as most attractive. Friedenberg and Bertamini (2015) found a similar patter using irregular 2D polygons. They varied the number of concave vertices and found a function similar to that in Phillips *et al*. (2010): Observers preferred either simple (no concavities) or complex shapes (four concavities). Note that the polygons with more concavities had also sharper angles compared to polygons with fewer concavities.

As mentioned earlier, Quinn *et al*. (1997) found evidence of a preference for curvature in 3‐ to 4‐month‐old infants. With respect to the role of threat and fear, it is relevant that the experience of fear is believed to emerge in children when they start to crawl or walk. This is because the exploration of the world brings more dangers. By around 7 months, infants discriminate fearful facial expressions (De Haan & Nelson, 1998; Lappänen & Nelson, 2009). In contrast, infants experience positive emotions and recognize happy facial expressions in the first days after birth (Vaish, Grossman, & Woodward, 2008). The result of the Quinn *et al*. study therefore is unlikely to be linked to a feeling of fear of angular shapes, although one could assume a more direct and instinctive response, which may not rely on feelings.

### Optimal stimulation of the visual system

Visual preference may depend on optimal stimulation of the visual system, and artists may select images that are tuned to the characteristics of the human visual system (Ramachandran & Hirstein, 1999; Zeki, 1992, 1999). It is therefore important to consider how the visual system processes curves and corners. In his seminal article, Attneave (1954) noted that, along contours, points of maximal curvature carry the greatest amount of information. De Winter and Wagemans (2008) found evidence to support this view: Participants were most likely to pick curvature maxima when asked to mark salient points along a contour (see also Norman, Phillips, & Ross, 2001). In general, a role for curvature extrema is well acknowledged in theories of shape representation (Cohen & Singh, 2007; De Winter & Wagemans, 2006, 2008; Feldman & Singh, 2005; Hoffman & Richards, 1984; Hoffman & Singh, 1997; Leyton, 1989). There is also evidence that attention is allocated preferentially to regions near corners (Bertamini, Helmy, & Bates, 2013; Cole, Skarratt, & Gellatly, 2007) perhaps because these locations are more informative.

If corners are important for shape analysis and attract attention, and if one assumes a preference for salient stimuli, one could predict a preference for angularity. This is the opposite of what the empirical studies have found. However, an alternative argument can be constructed. The Gestalt principles of grouping include proximity and good continuation (Wagemans *et al*., 2012). When oriented elements are aligned along a smooth path (collinearity), sensitivity in a contour detection task increases (Field, Hayes, & Hess, 1993). The stimuli used in these studies are known as *snakes* (similar local orientation) and *ladders* (orthogonal local orientation) (Bex, Simmers, & Dakin, 2001). What is relevant is that snakes are smooth paths. If it is easier to integrate collinear contours, this advantage applies to all locations on a smoothly curved contour. Although collinearity is present also along straight lines, angles are cases where contour integration may be harder.

We have considered whether a preference for curved shapes may be related to basic aspects of visual perception. Unfortunately, there is no clear pattern, as one could point to the importance of corners in shape analysis, as well as the importance of collinearity in contour integration.

### Relationship between curvature and complexity

Complexity has long been discussed in the empirical aesthetics literature. Birkhoff (1932) proposed that preference is inversely related to complexity, while Eysenck (1941) predicted that preference increases with complexity. A third option is that people have a preference for intermediate levels of complexity (Berlyne, 1970). Recently, Nadal, Munar, Marty, and Cela‐Conde (2010) analysed three different kinds of complexity: One relates to the amount and variety of elements, another relates to item organization, and the third to asymmetry. The authors suggest that each of these types of complexity modulates beauty ratings. Therefore, there are different ways to conceive, manipulate, and measure visual complexity.

How is curvature linked with perceived complexity? Silvia and Barona (2009) explored the relationship between curvature and perceived complexity while controlling for symmetry, prototypicality, and balance. They used circles and hexagons in one study, and irregular polygons in another. Because they collected judgments of complexity, they were able to test the link, and they found that the effect of curvature remained after controlling for appraisal of complexity. They also compared novices and experts, but the pattern for expertise was not straightforward: In the first study, an effect of curvature was present only for novices; in the second study, the effect was present only for experts.

In summary, based on the Silvia and Barona (2009) evidence, it seems unlikely that observers like curved shapes only because they are perceived as simpler. We will, however, revisit this question in our first experiment.

### Summary of experiments

Experiment 1 used abstract shapes. The aim was similar to that of Silvia and Barona (2009) including the fact that participants rated both preference and subjective complexity. The shapes were created as irregular polygons and smoothed versions of the same polygons so that the underlying parameters that determined the shapes were comparable.

In Experiment 2, shapes were placed on top of outdoor scenes. Stimuli were placed in the lower, the middle, or at the upper part of the scene. Participants indicated whether the object appeared to be within reach, and then performed a preference judgment. We reasoned that if angular shapes are associated with threat, then they should be even more disliked when they appeared near the observer.

Experiment 3 compared curved lines against not just angles but also straight lines. The idea was to see whether preference for smooth curves survives a comparison with stimuli that have no curvature and no corners (straight lines). The comparison between straight lines, which lack sharp angles, and angular lines tests the role of angles in visual preference.

Finally, Experiment 4 adopted the manikin task (De Houwer, Crombez, Baeyens, & Hermans, 2001; Krieglmeyer & Deutsch, 2010) as an implicit measure to examine approach and avoidance responses. The task consists of pressing the arrow keys to move a stick figure (i.e., the manikin) away or towards the stimulus. Participants identify with the manikin because the latter appears on screen after the participant presses a key (Krieglmeyer & Deutsch, 2010). Experiment 4 tested whether response times for angular shapes are faster when avoiding these shapes as compared to when approaching them. The threat hypothesis predicts a difference between these two responses. The hypothesis that people like curvature predicts that response times for curved shapes are faster for approach as compared to avoidance. These two patterns are not exclusive, and both effects may be confirmed.

## EXPERIMENT 1: CURVATURE, ARTICULATION, AND COMPLEXITY

The aim of Experiment 1 was to establish the preference for abstract shapes. Observers provided a rating for preference and for complexity. We used abstract meaningless shapes, and a new shape was presented in every trial, thus avoiding involvement of memory or familiarity. We predicted that curved shapes would be preferred compared to angular ones.

## Method

### Participants

Twenty participants took part (age range: 18–23; five left‐handed; 15 females). All participants had normal or corrected to normal vision. The experiment received ethics approval and was conducted in accordance with the Declaration of Helsinki (2008).

### Stimuli and apparatus

**Figure 3:**
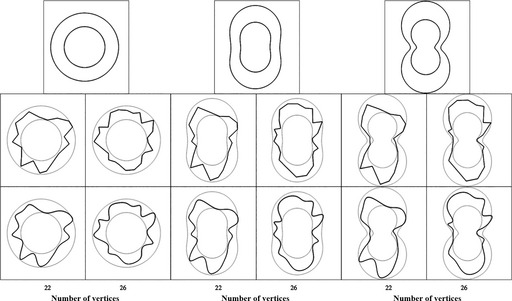
Experiment 1: Illustration of the stimuli. The examples at the top are the underlying functions. The small one has a value of *b* = 75 pixels, and for the large one, *b* = 125. From left to right, the value of a was 0%, 70%, and 90% of *b*. The relative difference between *a* and *b* changes a circle into a peanut shape. These basic functions were not presented to the observers. Instead, shapes were generated by selecting N vertices between the small and the large versions (i.e., with a random value of *b* between the two extremes). These vertices produced polygons like those shown in the second row. The third row shows the polygons after a cubic spline fit. On each trial, the stimulus presented was novel.

Stimuli consisted of irregular shapes with a black contour (Figure 3). Stimuli and experiment were created using Python and PsychoPy (Peirce, 2007). To generate polygons, points were sampled along a function. We chose the Cassini oval as the starting function because it can vary from circular to elongated to lobed.^1^ The Cassini ovals are a family of quadratic curves, defined as the points in the plane such that the product of the distances to two foci is constant. The parametric formula is:

x(i)=cos(i)√C

y(i)=sin(i)√C

where

$$C=a^{2}\text{cos}\left(2i\right)\;\pm \;\surd \left(b^{4}-a^{2}{\text{sin}}^{2}\left(2i\right)\right)$$

We chose the parameters *a* and *b* as follows: *b* was chosen randomly between 75 and 125 pixels. The top row in Figure 3 shows the versions with *b* = 75 and *b* = 125 inside each other. From left to right, there are three cases where *a* = 0 (cassini0), *a* = 70% of *b* (cassini70), and *a* = 90% of *b* (cassini90). These parameters change the basic shape from something close to a circle (cassini0) to an oval (cassini70) to a shape with two lobes (cassini90).

From these basic functions, we created polygons by choosing 22 or 26 vertices along the function, and by changing *b* and *a* for each vertex. Therefore, the vertices were confined to the region between the max and min sizes (top of Figure 3). For each polygon, we created a smoothed version by fitting a curve through the vertices using a cubic spline. Curved and angular stimuli can be compared by looking at the second and third rows in Figure 3. The combination of these factors generated shapes that varied in elongation and articulation. Stimuli were presented in one of three different orientations (0, 45, or −45°). Half of the trials presented angular contours, and half, curved contours. To exclude possible effects of memory and familiarity, each trial used a different stimulus. Participants sat at approximately 60 cm from the screen. Stimuli were presented on an Apple StudioDisplay 21” CRT monitor (1,280 × 1,024 at 60 Hz).

### Experimental design and procedure

**Figure 4:**
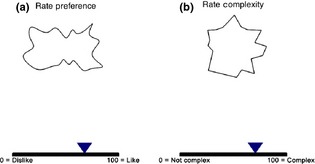
There were two tasks in Experiment 1. The Figure shows the response screen for (a) preference rating and (b) complexity rating.

A 2 × 3 × 2 × 3 within‐subjects design was employed with factors: Shape (angular vs. curved), Articulation (cassini0 vs. cassini70 vs. cassini90), Vertex (22 vs. 26), and Orientation (0, 45, and −45°). The experiment began with the instructions followed by practice (six trials). Each trial started with a fixation cross at the centre of a grey background for 500 ms, and then, the shape appeared and remained on screen until response. Intertrial time ranged between 0.6 and 1.6 s. The first task was to rate preference on a scale ranging from dislike (0) to like (100) with a mouse click, and the second task was to rate complexity from not complex (0) to complex (100) (Figure 4). The two tasks were performed in counterbalanced blocks.

The practice was followed by 180 experimental trials, which were identical to the practice except that novel shapes were presented. No stimulus was ever presented twice. The experiment lasted approximately 40 min.

### Analysis

A 2 × 3 × 2 repeated measures ANOVA was performed with Shape (angular, curved), Articulation (cassini0, cassini70 and cassini90), and Vertex (22, 26) as the within‐subjects factors. Preference and complexity ratings were the dependent variables. Orientation of the shapes was not entered in the analysis because it was only meant to increase variability. Moreover, because the stimuli were matched in a way that each shape was presented in its angular and curved version, we performed a correlation analysis between preference and complexity scores for each subject.

## Results

**Figure 5:**
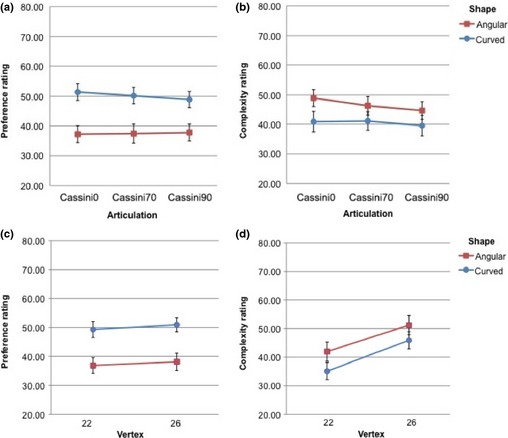
Results of Experiment 1. (a) Preference rating as a function of Articulation (cassini0 vs. cassini70 vs. cassini90) and Shape (angular vs. curved; separate lines). (b) Complexity rating as a function of Articulation (cassini0 vs. cassini70 vs. cassini90) and Shape (angular vs. curved; separate lines). (c) Preference rating as a function of Vertex and Shape (angular vs. curved; separate lines). (d) Complexity rating as a function of Articulation (cassini0 vs. cassini70 vs. cassini90) and Shape (angular vs. curved; separate lines). Error bars are SE of the mean.

The results are illustrated in Figure 5. The analyses on preference revealed a main effect of Shape, *F*(1, 19) = 14.96, *p *=* *.001, $\eta_{p}^{2}$ = .441: Curved shapes were preferred to angular ones (curved: *M* = 50.12, *SD* = 2.46; angular: *M* = 37.48, *SD* = 2.79). The other main effects and interactions were not significant (all *p*s > 0.2).

The analyses on complexity confirmed an effect of Shape, *F*(1, 19) = 8.76, *p *=* *.008, $\eta_{p}^{2}$ = .316, with angular shapes rated as more complex (*M* = 46.56; *SD* = 2.95) than curved ones (*M* = 40.48; *SD* = 3.29). The effects of Articulation, *F*(2, 38) = 5.53, *p *=* *.008, $\eta_{p}^{2}$ = .225, and Vertex, *F*(1, 19) = 121.08, *p *=* *.000, $\eta_{p}^{2}$ = .864, were also significant. Finally, the interaction effects were not significant with the exception of Shape × Articulation: *F*(2, 38) = 3.81, *p *=* *.031, $\eta_{p}^{2}$ = .167. The pattern for complexity was therefore different from the pattern for preference.

Data for preference and for complexity came from separate blocks, in which the same stimuli were shown in different random order. To compare the responses, we computed correlations. As complexity increased, scores for preference decreased slightly (*r* = −.041). However, this correlation was small and not consistent across subjects. For some subjects, the value was positive, and for some, negative. A *t*‐test (test value = 0) was not significant, *t*(19) = −0.425, *p *=* *.676. There was therefore no support for a link between the two judgments.

## Discussion

Experiment 1 confirmed that curved shapes were liked more than angular shapes. As the average rating was just above 50 for curved shapes and well below 50 for angular shapes, one may be tempted to say that observers displayed a dislike for angularity. However, as mentioned in the introduction, this interpretation needs caution because there is no baseline. Therefore, we can only rely on the relative difference between preference for angular and curved shapes.

With respect to complexity, angular shapes were judged as more complex than curved ones. As expected, an increase in number of vertices made shapes appear more complex. Shapes were rated as more complex when they were generated by the function that had no elongation (cassini0) rather than by more articulated functions (cassini70 or cassini90). This may be the consequence of greater ambiguity in orientation. Lack of a clear orientation can make shapes appear more complex. The fact that angular shapes were perceived as more complex is counterintuitive because a small set of vertices fully specifies a polygon. It appears that observers have a sense of what looks complex that is unrelated to amount of information necessary to encode the shape. With respect to the original aim of the study, what is important is that there was no correlation between preference and perceived complexity, in line with the conclusions in Silvia and Barona (2009).

## EXPERIMENT 2: CURVATURE AND PERIPERSONAL SPACE

Experiment 2 examined whether preference for curved shapes is modulated by perceived distance. According to Bar and Neta (2006), sharp angles are perceived as a threat and, in line with the approach–avoidance model (Panksepp, 2008), these shapes should be avoided. Experiment 2 was designed to test a specific implication of this view. A subset of shapes used in Experiment 1 were shown in one of three different locations on the screen (lower, middle and upper part). Because the background image was a scene with an extended surface layout, the shapes appeared at different distances. The task was to rate preference for each shape after judging whether the object was within reach. We expected that curved shapes would be preferred, but we reasoned that the effect would be stronger when shapes were presented near to the observer because any threat in near space is more important than a threat farther away.

## Method

### Participants

Twenty participants took part (age range: 18–50; 0 left‐handed; 13 females). All participants had normal or corrected to normal vision. The experiment received ethics approval and was conducted in accordance with the Declaration of Helsinki (2008).

### Stimuli and apparatus

**Figure 6:**
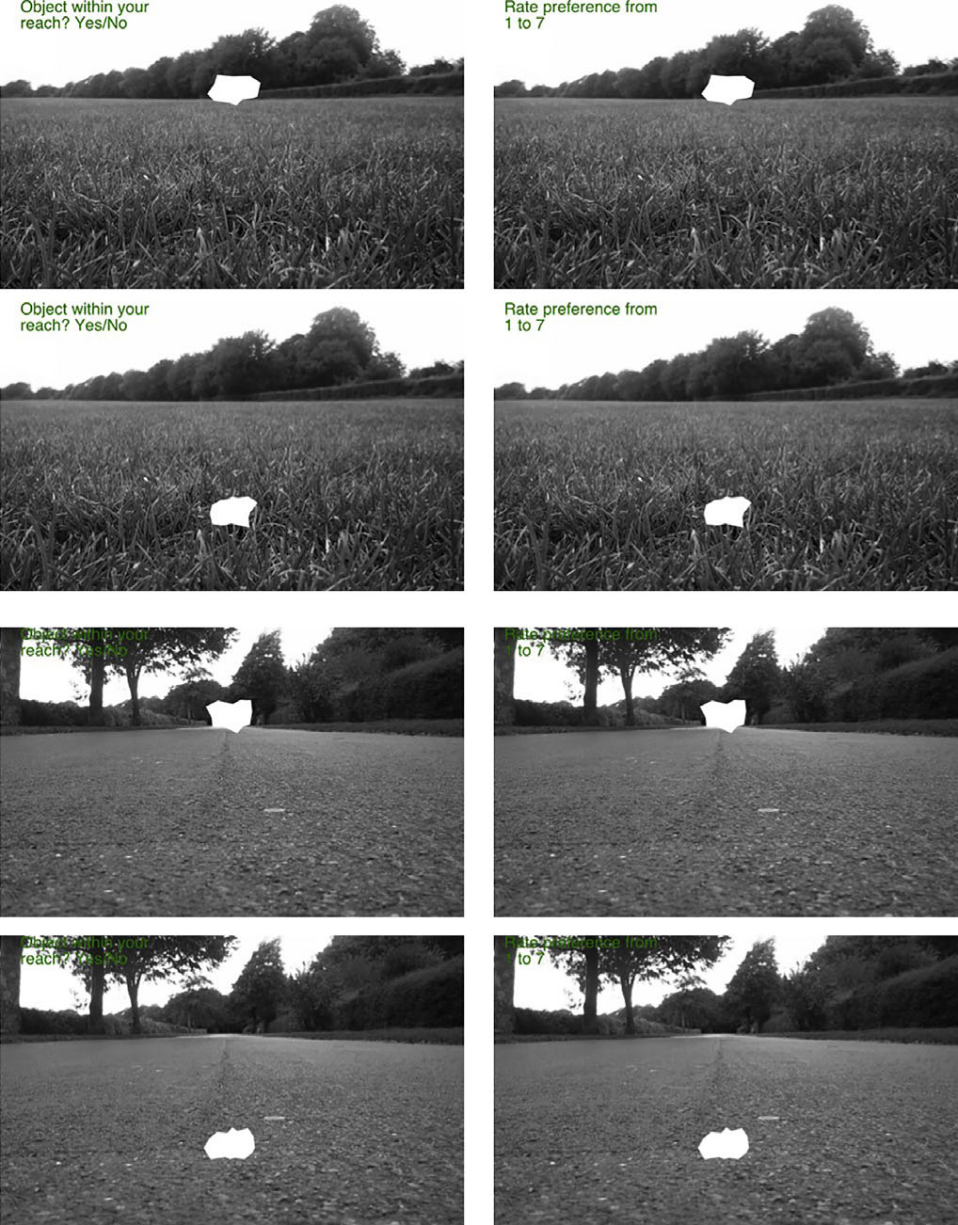
Experiment 2: Illustration of the stimuli and the two tasks. The first task was to judge whether the object was within reach (left column), and this task was followed by a preference rating (right column). The first two rows show the park scene, and the last two rows show the road scene. The first and third rows show the farthest location (Upper), and the second and fourth rows show the nearest location (Lower).

Stimuli consisted of white irregular abstract shapes with a curved or an angular contour. The stimuli were generated by the same algorithm described for Experiment 1, and they were all different from each other (half with an angular contour and half with a curved contour). However, in Experiment 2, we used only the 70% articulation (cassini70). These irregular shapes were presented over a background showing a country road, a grass field or a grey screen (control condition) (Figure 6). When the photos were taken, the camera was 10 cm above the ground. A marker was placed at 40 cm from the camera for the near location and at 640 cm for the far location. These markers were used to place the stimuli for the near and far conditions on screen, but were part of the normal texture of the scene and therefore almost invisible in the photographs. The intermediate position was at a location halfway between the other two.

Participants sat at approximately 60 cm from the screen. Stimuli were presented on an Apple StudioDisplay 21” CRT monitor (1,280 × 1,024 at 60 Hz).

### Experimental design and procedure

A 2 × 3 × 3 within‐subjects design was employed with factors: Shape (angular vs. curved), Location (lower vs. middle vs. upper), and Background (country road vs. grass field vs. grey screen). The experiment started with the instructions followed by the short practice (six trials). On each trial, the background image and the shape remained on screen until response. Participants performed two tasks: First they indicated whether the shape was at a reachable distance by pressing one of two keys labelled as Y (Yes) and N (No). Afterwards, they rated preference by pressing a number from 1 (low preference) to 7 (high preference).

After the practice, participants responded to 72 experimental trials, which were identical to the practice with the exception that novel shapes were presented. The experiment lasted approximately 30 min.

## Results

**Figure 7:**
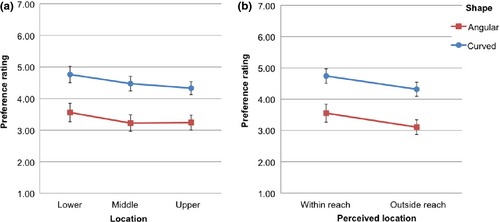
Results of Experiment 2. (a) Participants' preference rating as a function of Location (lower vs. middle vs. upper part of the picture) and Shape (angular vs. curved; separate lines). (b) Participants' preference rating as a function of Perceived location (within reach vs. outside reach) and Shape (angular vs. curved; separate lines). Error bars are SE of the mean.

The results are illustrated in Figure 7. We conducted two analyses because distance can be defined on the basis of where the shape was located (Actual Distance) or on the basis of what the participant reported (Perceived Distance). A 2 × 3 × 3 repeated measures ANOVA on actual distances showed an effect of Shape, *F*(1, 19) = 9.06, *p *=* *.007 $\eta_{p}^{2}$ = .323, confirming that curved shapes (*M* = 4.34; *SD* = 1.22) were preferred over angular ones (*M* = 3.45; *SD* = 1.32). None of the other main or interaction effects was significant (all *p*s > .05).

A 2 × 2 × 3 repeated measures ANOVA on Perceived Distance confirmed an effect of Shape, *F*(1, 19) = 9.39, *p *=* *.006, $\eta_{p}^{2}$ = .331. The effect of Location was also significant, *F*(1, 19) = 5.00, *p *=* *.037 $\eta_{p}^{2}$ = .208, meaning that participants overall preferred the shapes within reach (*M* = 4.08; *SD* = 1.39) versus out of reach (*M* = 3.67; *SD* = 1.18). None of the other main or interaction effects was significant (all *p*s > .05).

## Discussion

Experiment 2 confirmed that curved shapes are liked more than angular ones. Such preference was not modulated by the location on the screen or by perceived distance (within reach or outside reach). This is not consistent with the hypothesis that the preference for curvature is a by‐product of a negative response to angular shapes caused by a detection of threat, at least if one accepts the assumption that threat perception is modulated by distance. However, we have to be cautious with this conclusion as it is based on a null finding.

## EXPERIMENT 3: CURVATURE AND PLEASANTNESS

Experiment 3 investigated visual preference for stimuli that were even simpler than those used in Experiments 1 and 2. We used lines seen through an aperture so that there was no closed shape to form an object. To make patterns that could be sufficiently varied and interesting, we used seven lines seen within either a square or a circular aperture, and coloured each line with a different colour. We used straight lines with no angles, lines with an angle and therefore defined by three points, and parabolas fitted to the same three points. Note that in this study, we can compare curved stimuli against stimuli with straight lines that have no angles. If curvature is pleasant in itself, it should be preferred to straight lines even in the absence of angles. Conversely, if there is a dislike of angles, straight lines should be preferred to angular stimuli even in the absence of curvature.

## Method

### Participants

Fourteen observers took part (age range: 17–44; one left‐handed; eight females). All participants had normal or corrected to normal vision. The experiment received ethics approval and was conducted in accordance with the Declaration of Helsinki (2008).

### Stimuli and apparatus

Each stimulus consisted of a pattern of seven lines. There were three types of patterns: Straight, angular, and curved. The lines were seen against a black background and within an aperture. The shape of the aperture was a square (400 × 400 pixels) or a circle (400 pixels in diameter). The position of the lines was determined by selecting two points, one on the left side and one on the right side of a rectangular region. Given that the centre of the screen was the origin, the rectangle had coordinates between −180 and 180 vertically and between −200 and 200 horizontally. There was a constraint so that of the seven points at least three were in the upper half (between 1 and 180) and at least three were in the lower half (between −1 and −180). Each pair of points defined a line. To define an angular stimulus, one additional point was selected so that there was an angle on each of the seven lines. Horizontally this additional point was in a region between −100 and 100 pixels so that the angles were unlikely to be lined on top of each other. To define a curved stimulus, one additional point was selected with the same procedure and a parabola was drawn through the three points.

**Figure 8:**
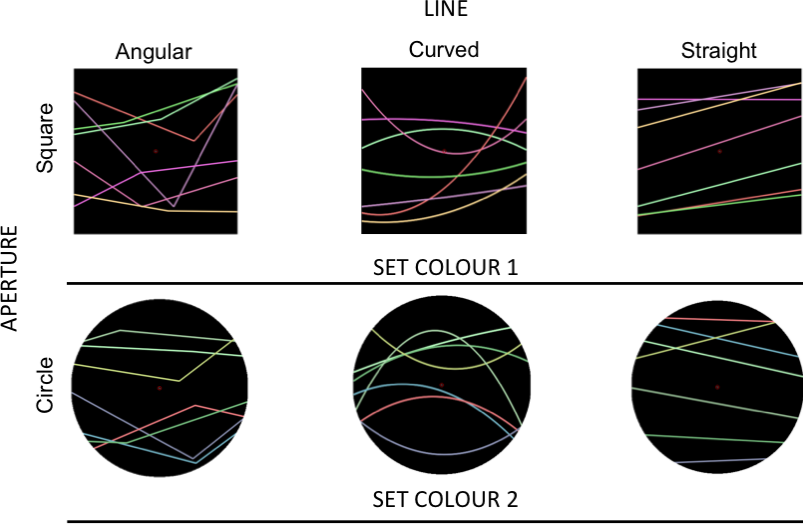
Experiment 3: Illustration of the stimuli. The top panel shows the pattern with angular, curved and straight lines, with the square aperture, and with Set colour 1. The bottom panel shows the pattern with angular, curved and straight lines, with the circular aperture, and with Set colour 2. All the patterns were generated with circle and square apertures and with both sets of colours.

The seven lines had different colours. In addition, there were two possible colour sets to make the patterns more variable and appealing (Figure 8). The stimuli and the experiment were created using Python and PsychoPy. Participants sat at approximately 60 cm from the screen. Stimuli were presented on a DELL M993s 19” CRT monitor (1,280 × 1,024 at 60 Hz).

### Experimental design and procedure

A 3 × 2 × 2 within‐subjects design was employed with factors: Line (angular vs. curved vs. straight), Aperture (squared vs. circle), and Colour (Set 1 vs. Set 2). The experiment started with the instructions followed by the practice (12 trials). Each trial started with a fixation cross for 500 ms. Then, the pattern appeared and remained on screen until response. The intertrial interval ranged between 0.6 and 1.6 s. Participants rated preference with a mouse click on a visual scale similar to the one used in Experiment 1 (from 0 = dislike to 100 = like; see Figure 4).

After the practice, participants saw 120 experimental trials, which were identical to the practice with the exception that novel patterns were presented. The experiment lasted approximately 20 min.

## Results

**Figure 9:**
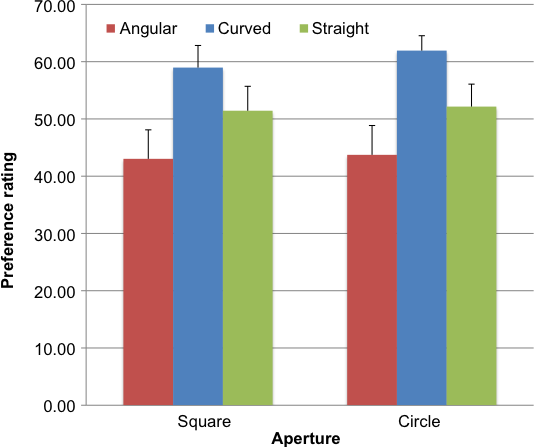
Results of Experiment 3. Participants' preference rating as a function of Aperture (square vs. circle) and Shape (angular vs. curved vs. straight; separate bars).

The results are illustrated in Figure 9. A repeated measures ANOVA on preference confirmed an effect of Line, *F*(2, 26) = 6.50, *p *=* *.005 $\eta_{p}^{2}$ = .333. The patterns with curved lines (*M* = 60.45; *SD* = 2.34) were preferred over the patterns with straight lines (*M* = 51.79; *SD* = 3.94), as confirmed by a paired samples *t*‐test, two‐tailed, *t*(13) = −2.41, *p *=* *.031, and over those with angular ones, *M* = 43.38; *SD* = 4.84), *t*(13) = −3.13, *p *=* *.008. Importantly, there was no difference on preference for patterns with angular lines versus those with straight lines, *t*(13) = 1.70, *p *=* *.114. There was no effect of Aperture, but there was an effect of Colour, *F*(1, 13) = 6.64, *p *=* *.023 $\eta_{p}^{2}$ = .338: Participants found Set 1 (*M* = 53.70; *SD* = 2.65) more attractive than Set 2 (*M* = 50.05; *SD* = 2.94). The interaction effects were not significant (*p*s > .05).

## Discussion

Experiment 3 revealed that curvature is preferred over angularity even for simple elements such as lines. Importantly, the presence of angles was not a key factor in generating a difference in preference scores. The stimuli with angles were disliked as much as those with straight lines and no angles. Therefore, this result is consistent with the hypothesis that curves have a pleasant appearance, which is not only a consequence of their lack of angularity.

It is true that in the comparison between the three conditions, the straight lines are simpler than either of the other two conditions. It was impossible to remove the angles without making the stimuli simpler. However, the results of Experiment 1 in combination with the results in Silvia and Barona (2009) suggest that perceived complexity is unlikely to be an important confound.

## EXPERIMENT 4: CURVATURE AND APPROACH BEHAVIOUR

In Experiment 4, we used the manikin task (De Houwer *et al*., 2001). Our previous experiments used explicit measure of preference, and it is important to compare those results with an implicit measure that is less affected by conscious strategies and expectations. This experiment will test whether perceiving angular shapes as threatening generate avoidance behaviour (Bar & Neta, 2006; Panksepp, 2008). Specifically, this task tests the tendency to approach curved shapes and to avoid angular shapes. As noted before, both tendencies may be confirmed. Therefore, unlike the previous three experiments, the manikin task is not a preference task. However, approach behaviour towards a stimulus can be seen as an indirect measure of preference.

Participants moved a stick figure towards or away from the stimulus. The rationale is that if the curved stimuli are attractive, then participants should move the manikin towards them faster than away from them. Conversely, if angular shapes signal threat, then participants should move the manikin away from them faster than towards them. Because of this hypothesis, we can divide trials into compatible (towards curved, away from angular) and incompatible (away from curved, towards angular).

## Method

### Participants

Thirty‐six participants took part (age range: 18–31; two left‐handed; 27 females). All participants had normal or corrected to normal vision. The experiment received ethics approval and was conducted in accordance with the Declaration of Helsinki (2008).

### Stimuli and apparatus

Stimuli were similar to those used in Experiment 1. Each shape was generated using 22 vertices starting from the cassini0 function. The manikin was a drawing of a stick figure consisting of a circle for the head and straight lines for the body and limbs. The manikin was 2.5 cm high and 1 cm wide. During movement, the right leg and the left leg became longer and shorter to generate the impression of walking. Participants sat at approximately 60 cm from the screen. Stimuli were presented on an Apple StudioDisplay 21” CRT monitor (1,280 × 1,024 at 60 Hz).

### Experimental design and procedure

**Figure 10:**
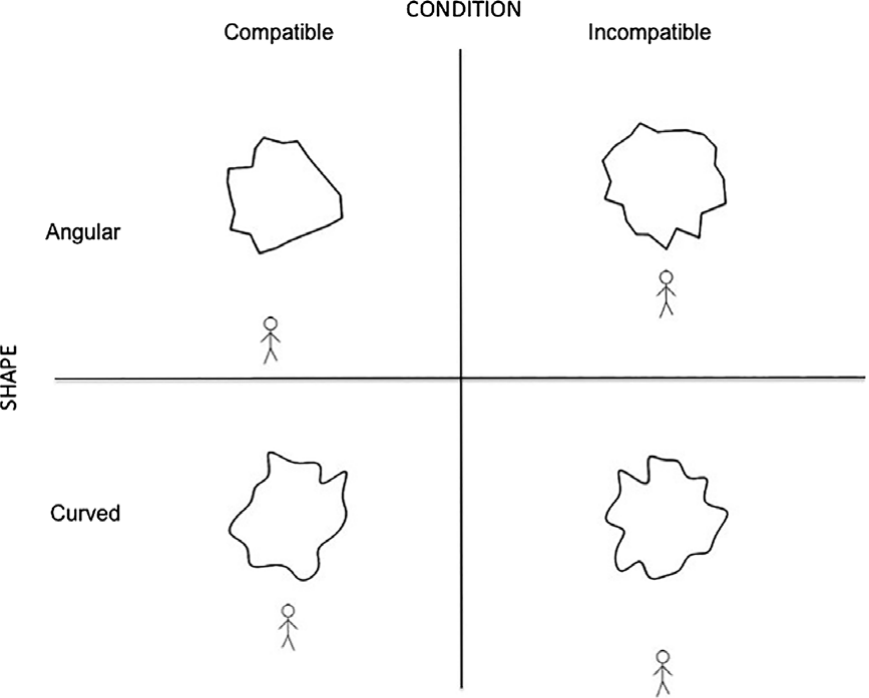
Experiment 4: Illustration of the design. The left panel shows the compatible task: The manikin was moved by the participant away from an angular shape (top) or towards a curved shape (bottom). The right panel shows the incompatible task: The manikin was moved by the participant towards an angular shape (top) and away from a curved shape (bottom). In this figure, the manikin is shown always underneath the stimulus, but it was presented above the stimulus with equal probability.

A 2 × 2 within‐subjects design was employed with factors: Condition (compatible vs. incompatible) and Shape (angular vs. curved). A trial started with a fixation cross. Participants were instructed to press 5 on the numeric pad to make the manikin appear on screen. After 750 ms, a shape (angular or curved) was presented at the centre of the screen. Depending on the condition, participants were instructed to identify with the manikin and move towards curved shapes and away from angular shapes (compatible condition), or vice versa (incompatible condition). The manikin could appear on the bottom or on the top of the screen. Participants moved the manikin by pressing 8 (upward) or 2 (downward) on the numeric pad. They were instructed to respond as fast and as accurately as possible. Depending on the initial position of the manikin (bottom or top) and direction (upward or downward), the figure stopped either at the edge of the screen or close to the shape (Figure 10). Three keypresses were needed to reach (38 pixels per step) the end position. The screen turned black 50 ms after the third keypress. The dependent variable was the time between the onset of the manikin and the last keypress. Participants completed one compatible block of 60 trials and one incompatible block of 60 trials. Each block was preceded by eight practice trials. The order of the compatible and incompatible blocks was counterbalanced across participants, and within a block, trials were presented in random order. The experiment lasted approximately 20 min.

### Analysis and data reduction

A 2 × 2 repeated measures ANOVA was performed for RT with Condition (compatible vs. incompatible) and Shape (angular vs. curved) as within‐subjects factors. RT was the time elapsed between the appearance of the manikin (first key) and its last displacement (last key). Only trials for which participants pressed the correct key were analysed. Seven participants were excluded from the analysis because they made errors in more than 25% of the trials, and consequently, it was impossible to compute the mean for each condition.

## Results

**Figure 11:**
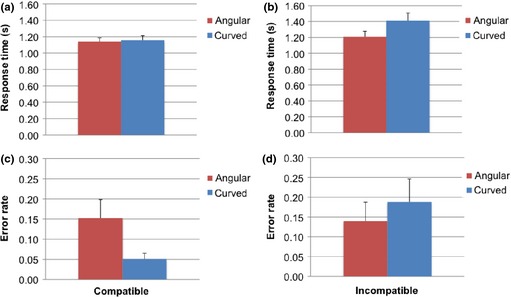
Results of Experiment 4. (a) Participants' RTs as a function of Condition (compatible vs. incompatible) and Shape (angular vs. curved; separate bars). (b) Error rates by Condition (compatible vs. incompatible) and Shape (angular vs. curved; separate bars). Error bars are SE of the mean.

The results are illustrated in Figure 11. The ANOVA on RTs confirmed an effect of Condition, *F*(1, 28) = 10.69, *p *=* *.003 $\eta_{p}^{2}$ = .276: Participants were faster in the compatible (*M* = 1.11; *SD* = 0.057) than in the incompatible trials (*M* = 1.27; *SD* = 0.088). The effect of Shape, *F*(1, 38) = 3.23, *p *=* *.051 $\eta_{p}^{2}$ = .145, was also significant: Faster responses for angular (*M* = 1.14; *SD* = 0.063) rather than curved shapes (*M* = 1.25; *SD* = 0.079). Importantly, there was an interaction between Condition and Shape, *F*(1, 28) = 18.22, *p *=* *.000 $\eta_{p}^{2}$ = .394, and therefore, this interaction has to be considered carefully to understand the results.

For angular stimuli, RTs did not differ between compatible and incompatible trials, *t*(28) = −1.68, *p *=* *.104. Participants did not move the manikin away from angular shapes faster (*M* = 1.14; *SD* = 0.24) than towards them (*M* = 1.21; *SD* = 0.37). In contrast, for curved stimuli, RTs differed between compatible and incompatible trials, *t*(28) = −3.93, *p *=* *.001. Participants moved the manikin closer to curved shapes faster (*M* = 1.16; *SD* = 0.29) than away from them (*M* = 1.41; *SD* = 0.52).

The pattern for the errors was consistent with that for RTs. The number of errors for angular stimuli did not differ between compatible and incompatible trials, *t*(35) = 0.192, *p *=* *.849, but for curved shapes, there were more errors in incompatible relative to compatible trials, *t*(35) = −3.19, *p *=* *.003. Therefore, participants made more errors when moving the manikin away from curved shapes than towards them.

## Discussion

Experiment 4 tested whether angular shapes generate an avoidance response. This hypothesis was not supported because response times did not differ for approach or avoidance. Responses to angular shapes were fast in both conditions (towards and away), and it is possible that these shapes attracted attention or were more arousing than the curved shapes. This result is in line with the corner enhancement effect (Cole *et al*., 2007).

We found that moving the manikin away from curved shapes was slower and more difficult (as participants made more errors in this condition) than moving it towards them, which is consistent with a preference to approach curved shapes. There was no avoidance for angular shapes but an approach response towards curved shapes. This result is problematic for the hypothesis that the aesthetic appeal of curved shapes derives from a dislike for angular shapes (Bar & Neta, 2007). Therefore, Experiment 4 reinforced the possibility that curved shapes are appreciated for their intrinsic properties.

## GENERAL DISCUSSION

There is empirical evidence showing a preference for curved stimuli over angular ones, but the source of such preference is under debate. The current study examined the nature of this preference and in particular whether smoothly curved contours are preferred because of how they are perceived, or indirectly because they are not angular.

In the introduction, we mentioned William Hogarth who claimed that curvature is directly linked with beauty because of the sense of variety that it expresses. In his book, Hogarth describes the waving line as the ‘line of beauty’ and the serpentine line as the ‘line of grace’ (Hogarth, 1753). Smoothly curved lines can also be seen as an example of good continuation in the Gestalt sense (Kanizsa, 1979). There are therefore reasons to believe that smoothly curved lines are liked because of their shape. On the other hand, we have to consider carefully the nature of angularity. Angular shapes are sometimes used in works of art (for instance in Mondrian's paintings), but for most people, they may be (automatically) associated with threat (Bar & Neta, 2006, 2007). Either a preference for curvature or a dislike for angularity may be sufficient to explain the curvature preference.

In Experiments 1, 2, and 4, we generated polygons by selecting points from the Cassini function. Starting from these polygons, we constructed shapes so that for each stimulus we had an angular polygon and a smoothed version, using a cubic spline. In Experiment 1, we collected judgments of preference and of complexity for the same stimuli. Curved shapes were preferred, and angular shapes were judged as more complex. This is counterintuitive as coding the shape of a polygon is simpler, as it relies on a small set of vertices compared to coding a smooth curve. It appears that observers relied on an impression of complexity that was affected by angularity. What is important for our study is the link between judgement of preference and judgement of complexity. No pattern emerged by comparing the two responses even though they were from the same observers and the same stimuli (in different blocks). In agreement with Silvia and Barona (2009), we suggest that preference for curvature is not closely related to degree of simplicity or complexity of the stimuli.

As discussed before, the curvature effect may derive from the fact that angular shapes signal a threat (Bar & Neta, 2006, 2007; Silvia & Barona, 2009). Experiment 2 tested one possible aspect of such hypothesis. Participants saw stimuli on the lower part (near) or on upper part (far) of a scene and reported whether they appeared within reach. After that, they provided a preference rating. We reasoned that angular shapes may be disliked more within peripersonal space as the threat is more imminent. The effect (the degree of preference for curvature) was unrelated to perceived distance. This was a null finding, but nevertheless it weakens the hypothesis of a threat association to angular shapes as the main explanation underlying the curvature effect.

Experiment 3 used patterns made of lines seen though an aperture. The introduction of straight lines in the design allowed us to assess the role of angles. People preferred curved lines over angular lines but also over straight lines. These results do not support the hypothesis that angles are critical in preference formation. There was no significant difference in preference for lines with and without angles.

Finally, Experiment 4 employed an implicit approach–avoidance measure. The manikin task consisted of pressing one key to move a figure towards a shape and another to move it away. There was no avoidance response for angularity but a strong approach response for curvature. This suggests that curved shapes contain properties that are enough to generate a visual preference and an approach response.

Taken together, our four experiments show a strong preference for curvature not linked to perceived complexity (Experiment 1), and the effect is not a direct consequence of the dislike for angularity (Experiment 2 and Experiment 3). The manikin task revealed indirectly that observers did not reject the angular shapes (Experiment 4) but displayed an approach response to smooth curvature. As mentioned in the introduction, angular elements are also present in many artworks, for example in Mondrian's compositions, which have aesthetic value. Preference formation is influenced by the combination of different factors, for instance the spatial relation between elements as expressed in Mondrian's artworks. Nevertheless, in the contexts that we have tested, we found that curvature was associated with a preference in naïve observers.

We still do not know the origin of this effect. It has been suggested that people tend to prefer what is easier to process, and that optimal stimulation of the visual system leads to a positive response. A possible example of this is bilateral symmetry because it is a type of regularity that the visual system is tuned to (Barlow & Reeves, 1979; Makin, Pecchinenda, & Bertamini, 2012a,b; Makin, Wilton, Pecchinenda, & Bertamini, 2012). However, if this was the mechanism underlying preference for curvature, then we should have found a link between preference and perceived complexity, but this link was not confirmed. Possibly preference for curvature might rely on subsequent analysis of visual structure. As such, curved shapes can be described as cases of good continuation (Wagemans *et al*., 2012) and perhaps they are good Gestalt. But the exact way in which grouping phenomena relate to the curvature effect is unclear. Our studies do not directly address this issue.

From an evolutionary perspective, a preference for round shapes may relate to neoteny, that is the retention in the adult of juvenile physical traits (less body hair, round head, flattened face, and round large eyes). There is evidence that neotenic traits are attractive for both men and women and have been subject to sexual selection (Gould, 1980; Penton‐Voak, Jacobson, & Trivers, 2004; Perrett *et al*., 1998). This idea could be the subject of future research.

### Conclusions

Most people prefer curved stimuli to angular stimuli. In our data, this effect was clear in all four experiments, including when lines were presented through an aperture and therefore did not form closed shapes. Moreover, our data suggest that the preference for curvature is not a by‐product of a negative response to angularity. We conclude that the curvature effect is likely to be caused by intrinsic characteristics of the stimuli, rather than what they might signal.

## Notes

1 The Cassini ovals, also known as Cassini ellipses, take their name from the astronomer Giovanni Domenico Cassini (1625–1712). He introduced them in 1680 and suggested that the orbit of the Sun was described by such function, with the Earth at one focus. The special case where *a* = *b* gives a figure of 8, known as a lemniscate. This was described by Bernoulli in 1694 without knowing about the earlier work by Cassini.
